# Development of a Dosage form for a Photoswitchable Local Anesthetic Ethercaine

**DOI:** 10.3390/ph16101398

**Published:** 2023-10-02

**Authors:** Alexey Noev, Natalia Morozova, Nikita Suvorov, Yuriy Vasil’ev, Andrei Pankratov, Mikhail Grin

**Affiliations:** 1 Department of Chemistry and Technology of Biologically Active Compounds, Medicinal and Organic Chemistry, Institute of Fine Chemical Technologies, MIREA-Russian Technological University, 86 Vernadsky Avenue, 119571 Moscow, Russia; suvorov.nv@gmail.com (N.S.); andreimnioi@yandex.ru (A.P.); michael_grin@mail.ru (M.G.); 2 P. Hertsen Moscow Oncology Research Institute — the Branch of the FSBI “National Medical Research Radiological Centre” of the Ministry of Health of the Russian Federation, 2nd Botkinsky pr. 3, 125284 Moscow, Russia; n.b.morozova@yandex.ru; 3 Department of Operative Surgery and Topographic Anatomy, I.M. Sechenov First Moscow State Medical University (Sechenov University), Trubetskaya St. Bldg. 8/2, 119435 Moscow, Russia; y_vasiliev@list.ru

**Keywords:** ethercaine, photopharmacology, local anesthetics, micelles, poorly water-soluble drugs

## Abstract

The toxicity of local anesthetics is a serious problem, given their widespread use. One of the main causes of the side effects of local anesthetics is their non-selectivity of action in the body. A possible way to increase the selectivity of the action of drugs is to use the photopharmacology approach. Previously, we described the light-controlled local anesthetic ethercaine, the biological effect of which can be controlled using light, thereby increasing its selectivity of action. An important limitation of ethercaine was its low solubility in water, limiting the potential of this compound. In this work, we developed a dosage form of ethercaine, which allowed us to increase its solubility from 0.6% to 2% or more. The resulting 1% solution of ethercaine hydrochloride in 4% Kolliphor ELP had high biological activity on the surface anesthesia model, while demonstrating low acute toxicity in mice with intravenous administration (4–5 times less than that of lidocaine).

## 1. Introduction

Pain is among the most common factors that deteriorate the quality of life of a modern person [1,2,3]. Different kinds of pain are distinguished depending on their locations, causes, and frequency, which affect the choice of drugs to attain the most efficient therapy. The main classes of painkillers are opioid and non-opioid drugs; the latter include non-steroidal anti-inflammatory drugs, antiepileptic drugs, antidepressants, local anesthetics, etc. [4]. Most of these drugs feature a number of side effects that range from mild local irritations and allergies to severe systemic complications, up to respiratory and cardiac arrest [5,6,7]. A number of strategies have been suggested to overcome the side effects of pain medications, including the development of highly selective agents that act on specific protein targets in the body; the use of nanostructured delivery forms, such as micelles, liposomes, and more complex structures; and the creation of agents that can be activated and controlled by external factors, for example, light [8,9,10,11,12]. To implement the latter strategy, photopharmacological approaches are applied [13,14,15,16], including the use of compounds of photoisomerizable classes, such as azobenzenes and spiropyrans. With the right choice of a molecule, one of its forms (the *E* or *Z* isomer in the case of azobenzene) exhibits biological activity, while the other form does not, where switching between these forms is performed using light. This feature of these compounds makes it possible to implement various modes of therapy that control the action of a drug after it is administered into the body. The use of a photoswitchable local anesthesia may increase intraoperative control over the depth and strength of nerve block in areas where conventional local anesthetics may lead to complications. Thus, light-controlled local anesthetics can be used in dentistry, maxillofacial surgery, and neurosurgery. Also, there are some topical procedures, including antimicrobial or anticancer photodynamic therapy and psoriasis UV-phototherapy, which can cause painful conditions [17,18]. Combining those procedures with light-activated local anesthetics can reduce pain. In addition, the reduced toxicity of these drugs in an inactive form may allow them to be administered systemically, with subsequent activation in areas of therapeutic interest to which the delivery of conventional local anesthetics is difficult or can cause side effects.

**Figure 1:**
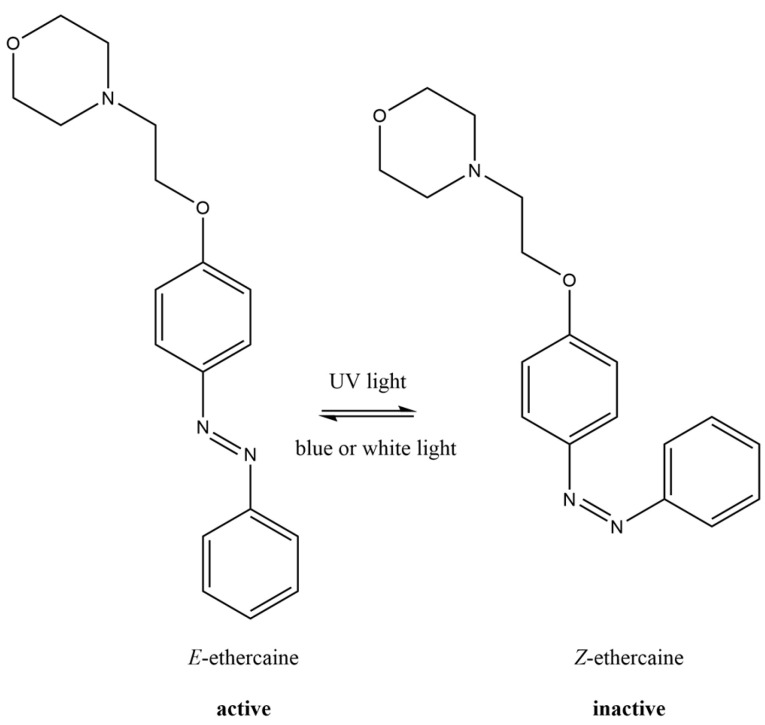
Isomerization of the ethercaine molecule.

Previously, we obtained a photoswitchable local anesthetic, ethercaine, and demonstrated the possibility of reversible light-controlled anesthesia in vivo using this compound [19]. When ethercaine solution was irradiated with blue or white light, local anesthetic properties of the compound appeared; however, after irradiation with UV light (365 or 395 nm), the biological activity disappeared almost completely (Figure 1).

This property makes it possible to reduce the number of side effects caused by the non-selective action of the drug in the body and provides a wide range of options for managing pain relief. However, despite the obvious advantages of ethercaine, its low solubility in water was one of its drawbacks. The use of excipients enabled an enhancement of its solubility to a limited extent but did not allow the full therapeutic potential of the substance to be revealed fully.

The purpose of this study was to select the optimal conditions for the solubilization of ethercaine and to perform a comparative analysis of the efficiency and acute toxicity of the potential drug.

## 2. Results and Discussion

### 2.1. Selection of Conditions for Ethercaine Solubilization

Poor water solubility is a common problem in the development of new drugs, which sometimes makes biological studies into new compounds and the further development of drugs on their basis impossible [20,21,22]. Many approaches to the solution of this problem have been suggested, including both the structural modification of compounds and the use of various delivery systems, such as micelles, liposomes, etc. [23,24,25,26,27,28].

**Figure 2:**
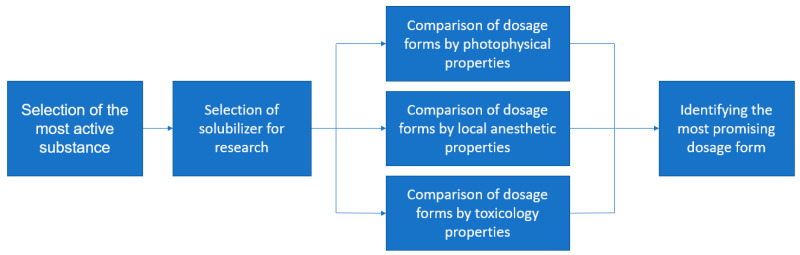
A schematic diagram of current research.

This work followed the research plan shown in Figure 2.

The most active substance was determined by us earlier [19]. Ethercaine (ETH), a light-driven local anesthetic, has limited water solubility due to the presence of a hydrophobic azobenzene moiety. To solve the solubility problem, we previously obtained a micellar solution of ETH in 4% Kolliphor ELP, a solubilizer approved for clinical use [19]. This dosage form was used to study the local anesthetic activity on the cornea of a rabbit eye. However, the maximum concentration of ETH in 4% Kolliphor ELP, such that the solution remained stable, was 0.6 wt.%. This concentration does not allow the drug to achieve the maximum possible activity; in addition, the concentration recommended for screening local anesthetics in the model of surface anesthesia in vivo is 1% [29].

In view of this, various solubilization methods were compared to identify the optimal conditions for biological studies and potential clinical applications.

Since ETH itself, as a free base, is virtually insoluble in water and only partially soluble in 4% solution of Kolliphor ELP, at the first stage it was decided to study the effects of increasing the concentration of Kolliphor ELP and replacing it with Pluronic F127. Pluronic F127 is also an FDA-approved solubilizer of a different nature to the Kolliphor ELP, so it was selected as an alternative solubilizer for the study. Both substances are surfactants capable of forming direct micelles in aqueous solutions for loading hydrophobic compounds that are sparingly soluble in water. We chose a 4% concentration of Kolliphor ELP as the initial concentration that is suitable for the solubilization of hydrophobic dyes but is safe for clinical use, including for intravenous administration [30,31,32]. In the case of other administration routes, for example, for ophthalmic use, this concentration can be increased [33]. For further studies, we chose a 10% concentration of Kolliphor ELP. Despite an almost two-fold increase in the maximum solubility of ETH (Table 1) with an increase in the concentration of Kolliphor ELP from 4% to 10%, data on biological activity indicated an unstable and undulating local anesthetic effect on the cornea of the rabbit eye according to the Regnier method, which appeared to be due to the delayed drug release at high surfactant concentrations. The use of another solubilizer, Pluronic F127, did not improve the situation significantly.

**Figure 3:**
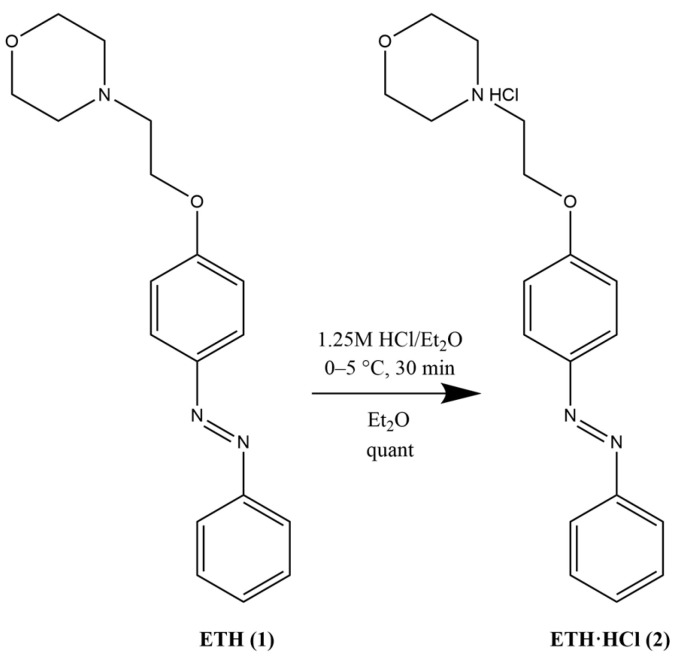
Preparation of ethercaine hydrochloride (ETH·HCl, **2**).

The majority of local anesthetics (LA) contain a tertiary nitrogen atom in the structure, which is necessary for the manifestation of biological activity. The most common dosage form of LA are aqueous solutions of their salts, mainly hydrochlorides. This form was also chosen to study the solubilization of ethercaine. One of the common methods for preparing amine hydrochlorides is their treatment with HCl solution in organic solvents. One such solvent may be diethyl ether [34]. A method for the preparation of ethercaine hydrochloride (ETH·HCl) using HCl/Et_2_O, which is also suitable for its derivatives, was developed and optimized (Figure 3).

The ETH·HCl salt obtained was characterized using various physicochemical methods of analysis (^1^H and ^13^C NMR spectroscopy, mass spectrometry, and UV spectrophotometry). Full NMR spectra and LC-MS data for the obtained compounds are provided in the Supplementary Materials (Figures S1–S7).

**Figure 4:**
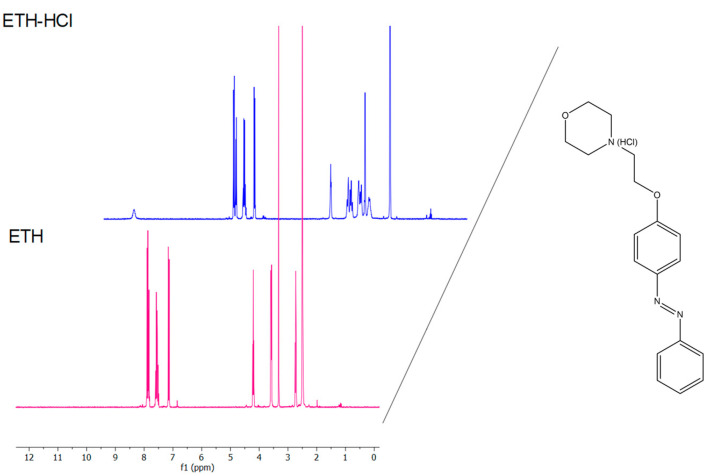
Superposition of the ^1^H NMR spectra of ethercaine as the hydrochloride (ETH·HCl, compound **2**) and the free amine (ETH, compound **1**).

^1^H NMR spectroscopy showed that a signal from an additional proton in ETH·HCl relative to the free amine in ETH appeared, and the chemical shifts of the protons of the morpholine ring and the ethoxy linker changed (Figure 4).

The salt **2** obtained featured a significantly higher solubility in water without the use of auxiliary compounds, similar to that of ETH in 4% Kolliphor ELP, although still below the optimal concentration in 1% Kolliphor ELP solution.

At the next stage of the solubility study, micelle-forming solubilizers and an anesthetic in the form of the ETH·HCl salt were used. As a result, ETH·HCl solutions with concentrations of at least 20 mg/mL in 4% Kolliphor ELP and 4% Pluronic F127 were obtained.

Table 1 presents the data obtained by optimizing the solubilization conditions of ethercaine.

The data obtained on the solubilization of ETH show that the conditions were found under which drug concentrations relevant for both its experimental and clinical use can be achieved.

### 2.2. Spectrophotometric Determination of ETH·HCl Z→E Half-Transformation Time ${(t}_{1/2})$ in Solutions

A key characteristic of photopharmacological agents is the time of the half-transformation of the less stable *Z*-form obtained using irradiation, back to the *E*-form. In the case of ethercaine, the *Z*→*E* half-transformation time determines the time during which ethercaine remains in an inactive form, which prevents its side effects. This parameter depends on a number of conditions, including the solvent; therefore, at the next stage, the effect of the solubilizer on the $t_{1/2}$ value was examined. The study was carried out by a standard method using UV/Vis spectrophotometry. At the beginning of the experiment, the spectrum of the initial photostationary state (PSS) with predominance of the *E*-form was recorded, after which the solution was irradiated with UV light (λ = 365 nm) and the spectrum of the resulting PSS, with predominance of the Z-form, was recorded.

**Figure 5:**
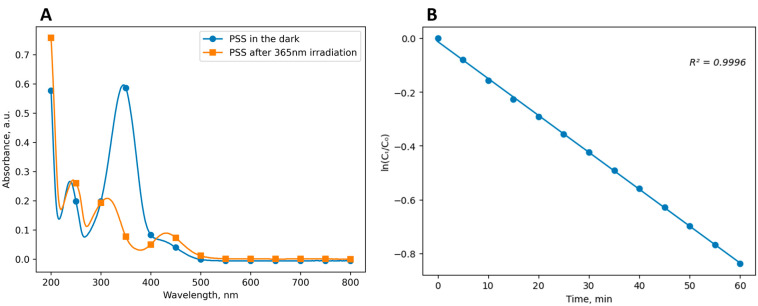
(**A**) Absorption spectra of an ETH·HCl solution in Kolliphor ELP in PSS with predominance of the *E* (in the dark) and *Z* (after irradiation with 365 nm light) isomers. (**B**) Time dependence of ln(C_t_/C_0_) plotted in linearized coordinates.

Next, the rate of reverse isomerization was monitored by measuring the absorption intensity at 5 min intervals. Figure 5A displays the representative absorption spectra of the ETH·HCl solutions in 4% Kolliphor ELP in PSS with predominance of the *E* and *Z* isomers. Figure 5B shows the time dependence of the absorption intensity for an ETH·HCl solution in 4% Kolliphor ELP, plotted in linearized coordinates. The linear correlation (R^2^ = 0.9996) indicates the first order of the reaction, which is consistent with the published data for azobenzene isomerization reactions.

Gradual conversion of *Z*-ethercaine hydrochloride back to the *E*-form was observed during the experiment. The calculated values of $t_{1/2}$ are shown in Table 2.

The results obtained indicate that the *Z*-isomer is fairly stable in both solubilizers.

### 2.3. Comparison of the Local Anesthetic Activity of 1% Solution of ETH·HCl in 4% Solutions of Kolliphor ELP and Pluronic F127

Further, the local anesthetic activity of ETH·HCl in 4% Kolliphor ELP and 4% Pluronic F127 was studied. Possible ethercaine applications include light-controlled pain relief during painful topical procedures in dermatology, neurosurgery, photodynamic therapy, etc., so the topical local anesthesia model was used in the biological activity assessment. One of the most convenient local anesthesia models is on rabbit cornea [19,35]. The essence of the method is to estimate the number of eye closures during the tactile exposure of the cornea of a rabbit’s eye. According to the method, four animals (eight experimental points) were selected for each group, except two groups with a negative control. For the latter two animals (four experimental points) were used due to the lack of the activity and to minimize the number of animals in the experiment. The animals were randomly divided into four experimental (1% ETH·HCl in 4% Kolliphor ELP or in 4% Pluronic F127, two modes with and without UV irradiation) and four control groups (4% Kolliphor ELP or 4% Pluronic F127, two modes with and without UV irradiation). Data for the 2% lidocaine hydrochloride and 0.6% ETH in 4% Kolliphor ELP were obtained before using identical conditions [19].

Considering the above values of the half-transformation time of ETH·HCl in 4% Kolliphor ELP and 4% Pluronic F127 ($t_{1/2}$ = 51 min and 69 min, respectively), we assumed that a single irradiation of the drug solution with light with λ = 365 nm immediately before the administration would make the exposure of a living organism to UV light unnecessary.

A quantitative measure of the strength of local anesthesia in this method was the Regnier index, a detailed description of which is given in the Section 3. The values of the local anesthetic activity obtained in this work are shown in Table 3, along with the data that we published previously.

The values obtained exhibited a significant non-linear increase in the strength of the local anesthetic effect of 1% ETH·HCl solutions in 4% Kolliphor ELP and 4% Pluronic F127, compared with the data obtained previously for 0.6% ETH in 4% Kolliphor ELP (the Regnier indices were 644 ± 30, 656 ± 18, and 232 ± 50, respectively). In addition, the strength of the local anesthetic effect at a given concentration of ethercaine was superior to that of the 2% lidocaine hydrochloride used as a positive control. If ETH·HCl solutions are irradiated with UV light (λ = 365 nm), a significant (by a factor of 16–20) decrease in the local anesthetic effect occurs, which indicates that local anesthesia can be successfully controlled by light.

The comparison of 1% solutions of ETH·HCl in 4% Kolliphor ELP and 4% Pluronic F127 showed that the Regnier indices obtained using the two different solubilizers did not differ statistically.

### 2.4. Examination of Ethercaine Acute Toxicity in 4% Solutions Kolliphor ELP and Pluronic F127

One of the important factors in the choice of excipients used in a dosage form is the toxicity exhibited by the resulting formulation. Aqueous solutions of local anesthetics are known to feature a relatively high toxicity and, should they enter the systemic circulation, can cause side effects up to respiratory and cardiac arrest, which, in the absence of immediate medical attention, can lead to death [5,6,36]. Side effects caused by local anesthetics primarily have the form of acute toxicity; therefore, the median lethal dose, LD50, was used to compare the toxicity of the experimental formulations. The experimental animals used in these studies are typically rats and mice, and the latter were chosen in this work. The rapid study of acute toxicity during intravenous administration in mice is possible using the probit method in Prozorovsky’s modification [37]. Four experimental groups were randomly formed (1 group per dose), with two animals per group.

**Figure 6:**
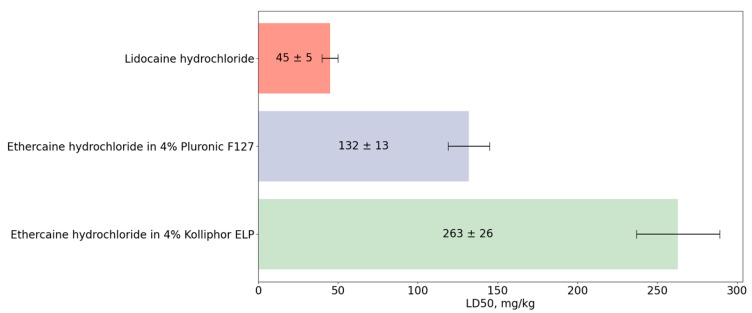
Acute toxicity of lidocaine and ethercaine in solutions of various solubilizers.

Lidocaine, a local anesthetic widely used in clinical practice, was chosen as the reference drug whose LD50 value was determined as 45 ± 5 mg/kg. It was found that micellar solutions of ethercaine are three-to-six-times less toxic when administered intravenously than an aqueous solution of lidocaine (Figure 6).

The lower toxicity of ethercaine in both dosage forms compared to lidocaine is undoubtedly an advantage; it is comparable to that of fomocaine, which is a close structural analogue of ethercaine but does not have its photoswitching property [38].

It was found that a solution of ethercaine hydrochloride based on 4% Kolliphor ELP is two-times less toxic when administered intravenously than a similar solution in 4% Pluronic F127 (LD50 was 263 ± 26 mg/kg and 132 ± 13 mg/kg, respectively). It is known that the intravenous administration of lipid emulsions can reduce or completely stop the manifestations of acute toxicity of local anesthetics [39,40]. This effect may explain the observed difference between the two solubilizers, since Kolliphor ELP is of a lipid nature, unlike Pluronic F127.

## 3. Materials and Methods

### 3.1. Materials

All the chemicals were obtained from commercial sources (Merck KGaA, Darmstadt, Germany; Acros Organics—part of Thermo Fischer Scientific, Waltham, MA, USA). Kolliphor^®^ ELP was obtained from BASF, Ludwigshafen, Germany. DMSO-d6 was obtained commercially from Cambridge Isotope Laboratories, Inc. (Tewksbury, MA, USA). Silica gel 60 (Merck KGaA, Darmstadt, Germany) was used for column chromatography. Analytical TLC was performed on aluminum plates with F245 silica gel 60 (Merck KGaA, Darmstadt, Germany). Lidocaine hydrochloride solution (20 mg/mL, Borimed, Borisov, Belarus) was obtained commercially and used without any additional preparation.

UV/Vis spectra were obtained using a Shimadzu UV1800 UV/VIS spectrophotometer (Shimadzu Corporation, Kyoto, Japan) in a 10 mm thick quartz cell. LCMS data were recorded on Agilent 6160 (Agilent, Singapore) with electrospray ionization either in a positive or negative mode. NMR spectra were obtained on a Bruker DPX300 spectrometer (Bruker Corporation, Billerica, MA, USA) using DMSO-d6 as solvent. Residual solvent was used as the reference standard for spectra calibrating.

Non-anesthetized mature male rabbits of the Soviet chinchilla breed weighing 2–3 kg, obtained from the KrolInfo farm (Orekhovo-Zuyevo, Russia), were used in this study. Animals were kept under standard conditions (humidity 50–60%, temperature 19–22 °C). A 12 h lighting cycle was maintained in the animal premises, where each animal was kept in a separate cage. Animals were given ad libitum access to standard extruded feed stuff for rabbits “CHARA” (CJSC “Assortiment-Agro”) and clean drinking water. Water treatment was performed using a “7 TECHNOCOM” block modular system (LLC “7 TECH”). For in vivo surface anesthesia measurements, an in-house anesthesiometer was made, with a fixed length of a nylon filament (l = 1.0 cm, d = 0.125 mm).

Statistical analysis was performed and figures were plotted using Python-programming-language standard functions and Matplotlib package for Python [41]. All biological data conformed to a normal distribution (Shapiro–Wilk’s W test, *p* > 0.05). All data are expressed as the mean ± standard deviation.

### 3.2. Chemistry

#### 3.2.1. Synthesis of 4-(2-(*N*-Morpholino)-Ethoxy)-Azobenzene Hydrochloride (Ethercaine Hydrochloride, **2**)

4-(2-(*N*-Morpholino)-ethoxy)-azobenzene (1) (1.0 g, 3.21 mmol) was dissolved in diethyl ether (10 mL) at 0–5 °C. An HCl solution in diethyl ether (6.0 mL, 1.25 M) was added and the mixture was stirred for 1 h. After that, the product was concentrated and dried in vacuo. The product **2** (pale-yellow powder) was obtained with a quantitative yield.

^1^H NMR spectrum of compound **2** (300 MHz, DMSO-d6) δ, ppm: 11.40 (br. s, 1H, ·HCl); 7.88 (m, 4H, 4×CH); 7.57 (m, 3H, 3×CH); 7.22 (m, 2H, 2×CH); 4.56 (m, 2H, 1×CH_2_); 3.92 (m, 4H, 2×CH_2_); 3.54 (m, 4H, 2×CH_2_); 3.22 (m, 2H, 1×CH_2_).

^13^C NMR spectrum of compound **2** (75 MHz, DMSO-d6) δ, ppm: 51.6, 54.7, 62.6, 63.1, 115.3, 122.2, 124.5, 129.4, 130.9, 146.6, 151.9, 160.1.

MS (ESI^+^) m/z: [M-HCl+H]^+^, calculated for (C_18_H_22_N_3_O_2_)^+^ 312.2, found 312.2 (100%).

#### 3.2.2. Preparation of a Micellar Solutions of 4-(2-(*N*-Morpholino)-Ethoxy)-Azobenzene Hydrochloride (**2**)

A solution of 4-(2-(*N*-morpholino)-ethoxy)-azobenzene hydrochloride (**2**) (50.0 mg) in dichloromethane (2 mL) was added dropwise to a freshly prepared 4% aqueous solution of solubilizer (Kolliphor^®^ ELP or Pluronic F127) (5 mL) heated to 45 °C with continuous argon bubbling. Bubbling was continued until strong foaming started. A clear orange solution with a concentration of 1.0% was obtained, which was subsequently filtered in turn through 0.45 µm PTFE filter and then through 0.22 µm PTFE filter.

### 3.3. Investigation of Photophysical Properties 

UV/Vis spectroscopy was used for the time determination of *Z*-isomers’ half-lifes. Solutions were kept in the dark at room temperature before the experiment. First, the spectrum for ${PSS}_{dark}$ was registered, which corresponded to the PSS with *E*-isomer prevalence. Then, the solution was irradiated with UV light (λ = 365 nm) for 5 min to give the PSS with *Z*-isomer prevalence. The spectrum for ${PSS}_{365nm}$ was registered. After that, back *Z*-*E* isomerization was observed at $\lambda_{\max\left(E\right)}$ for 60 min in increments of 5 min. Then, $\ln\frac{A_{t}}{A_{0}}$ values were calculated using Equation (1):

(1) $$\ln\frac{A_{t}}{A_{0}}=\ln\frac{{Abs}_{PSS(dark)}-{Abs}_{time}}{{Abs}_{PSS(dark)}-{Abs}_{PSS(365nm)}},$$

where ${Abs}_{PSS(dark)}$, ${Abs}_{PSS(365nm)},$ and ${Abs}_{time}$ are absorbance values at $\lambda_{\max\left(E\right)}$ in the dark at the beginning of the experiment, after UV light (λ = 365 nm) irradiation and at different time points from 0 to 60 min, respectively. Then, a graph was plotted depending on the $\ln\frac{A_{t}}{A_{0}}$ values from the time, a trend line was calculated and a first-order rate constant (*k*) for the thermal back *Z*–*E* isomerization reaction was obtained as the slope of the trend line.

For a first-order reaction, the half-life $t_{1/2}$ can be calculated using Equation (2):

(2) $$t_{1/2}=\frac{\ln2}{k}$$

### 3.4. Biology

All manipulations with animals were approved by the Committee of National Medical Research Radiological Centre of the Ministry of Health of the Russian Federation for bioethical control over the maintenance and use of laboratory animals for scientific purposes (Minutes No. 31 dated 15.12.2022), and performed in accordance with the national and international rules for the humane treatment of animals (European Convention for the Protection of Vertebrate Animals Used for Experimental and Other Scientific Purposes, Council of Europe (ETS 123), Eighth Edition of the Guide for the Care and Use of Laboratory Animals (NRC 2011)) [42]. All materials, methods and experimental procedures where animals were used are described in accordance with ARRIVE rules [43].

#### 3.4.1. Preparation of Animals and Arrangement of Groups

For determination of the sensitivity of rabbit eye cornea using the surface anesthesia method, a total of four rabbits with a basic sensitivity threshold of 1–2 touches were selected. Animals were used repeatedly, and the minimum interval between experiments was 7 days. Each eye was considered as a separate experimental point. Four test groups (the test solution with and without irradiation for each of the 2 test solutions) and four control groups (the control solution with and without irradiation for each of the 2 solutions: 4% aqueous solution of Kolliphor^®^ ELP and 4% aqueous solution of Pluronic F127, which were chosen as a negative control) were used. Four animals were used in each of the experimental groups (*n* = 8). Two animals were used in each of the negative control groups (*n* = 4) due to the expected lack of effect and to minimize the number of animals in the experiment. Animals were randomly divided into groups.

For determination of the acute toxicity, male C57Bl/6×CBA mice (19–26 g) were used. The animals had access to food and water ad libitum and were maintained at 24 ± 2 °C with a 12 h light/dark cycle. Four experimental groups of two animals per group (dose) were used.

#### 3.4.2. Irradiation Modes

Experiments with animals were carried out in a dark room with totally shaded windows. Since experiments cannot be performed in total darkness, a 50 W LED light source with a wavelength of 625 nm, which lies outside the absorption bands of the UV/Vis spectrum of the test compound, was used for illumination. It was arranged at a maximum distance from an animal and faced in the opposite direction. Before the start of the experiments with light exposure, solutions of the compounds being studied were preliminarily irradiated for 5 min with light at a wavelength of 365 nm (LED flashlight, 0.5 W).

#### 3.4.3. Determination of the Sensitivity of Rabbit Eye Cornea Using the Surface Anesthesia Method

The local anesthetic effect was determined using the rabbit-cornea model as described [19]. Before the experiment began, the animal was restrained, leaving only its head free. The eyelashes were cut and the sensitivity threshold was determined for each eye. The test solution (2 × 0.2 mL) was instilled into each conjunctival sac using a syringe without a needle. After that, the sensitivity threshold of the eye cornea to tactile impact was determined. The first determination was performed at the 8th minute of the experiment and then at the 10th, 12th, 15th, 25th, 30th, 35th, 40th, 45th, 50th, 55th and 60th minutes (13 values overall). Each time, tactile stimulation of the same strength and rhythm was applied to the cornea and the minimum number of touches causing the eyelids to close (but no more than 100 touches and no longer than for 1 min) was recorded. The Regnier index was calculated as the total of 13 values within 60 min using Equation (3):

(3) $$\mathrm{Regnier}\;\mathrm{index}\;\left(RI\right)=\sum_{i=1}^{13}n_{i}$$

where *n* is the number of touches before the eyelids closed.

Thus, the Regnier index ranges from 13 (complete absence of anesthesia) to 1300 (complete anesthesia for 1 h).

#### 3.4.4. Determination of the Acute Toxicity

The acute toxicity was measured using the Prozorovsky method [33] in mice using an intravenous route of administration. In this method, 4 groups of 2 animals each were used, all of them were administered the study drug. In each group, the dose of the drug was different; the 4 closest doses described in [33] were used. After intravenous administration, the animals were observed for 2 weeks. The quantitative assessment was the number of dead animals in each group after the observation period. After this, according to the tables given in [33], the corresponding values of LD50 and errors were found.

## 4. Conclusions

The hydrochloride form of the light-controlled local anesthetic ethercaine was obtained, with the aim of the better water solubility of the molecule. Its solubilization was examined and dosage forms based on micellar solutions of Kolliphor ELP and Pluronic F127 were obtained, which made it possible to increase the solubility of the anesthetic in water significantly, from 0.6 to 2 wt.% or more. Ethercaine solutions were studied using two solubilizers of different natures; their photophysical, biological, and toxicological properties were examined. The half-life values of the *Z*-form of ethercaine in solutions of Kolliphor ELP and Pluronic F127, as well as their local anesthetic properties in vivo, were similar, but a significant difference was found when studying their toxicity when administered intravenously in mice. Taking the results obtained into account, the dosage form of ethercaine hydrochloride, which is a 1% micellar solution of anesthetic in Kolliphor ELP, is the most optimal form both for experimental studies and for potential clinical applications.

## Figures and Tables

**Table 1:** Studies of the solubilization conditions of ethercaine.

Chemical Form of Ethercaine	Excipients	Maximum Solubility of Ethercaine, mg/mL
ETH	-	<1
ETH	4% micellar solution of Kolliphor ELP	6
ETH	10% micellar solution of Kolliphor ELP	10
ETH	4% micellar solution of Pluronic F127	7
ETH·HCl	-	6
ETH·HCl	4% micellar solution of Kolliphor ELP	>20
ETH·HCl	4% micellar solution of Pluronic F127	>20

**Table 2:** *E→Z* half-transformation times of ethercaine hydrochloride in solutions of solubilizers.

Solubilizer	Half-Transformation Time ($t_{1/2}$), Min
4% micellar solution of Kolliphor ELP	51
4% micellar solution of Pluronic F127	69

**Table 3:** Local anesthetic activity of ethercaine on rabbit eye cornea according to the Regnier method.

Compound	Regnier Index (Min—13, Max—1300)	
	Dark	UV λ = 365 nm
4% Kolliphor^®^ ELP	13 (*n* = 4)	13 (*n* = 4)
4% Pluronic F127	13 (*n* = 4)	13 (*n* = 4)
2% lidocaine hydrochloride	451 ± 40 (*n* = 8) ^a^	469 ± 37 (*n* = 8) ^a^
0.6% ETH in 4% Kolliphor ELP ^a^	232 ± 50 (*n* = 8) ^a^	22 ± 3 (*n* = 8) ^a^
1% ETH·HCl in 4% Kolliphor ELP	644 ± 30 (*n* = 8)	32 ± 16 (*n* = 8)
1% ETH·HCl in 4% Pluronic F127	656 ± 18 (*n* = 8)	40 ± 11 (*n* = 8)

^a^ the values were obtained earlier [19].

## Data Availability

The data presented in this study are available from the authors on request.

## Supplementary Materials

The following supporting information can be downloaded at: https://www.mdpi.com/article/10.3390/ph16101398/s1, Figures S1–S4: NMR spectra of the compounds; Figures S5–S7: LC-MS data for the ethercaine hydrochloride.
