# Supplementary material for: Development of a Dosage form for a Photoswitchable Local Anesthetic Ethercaine
Source: Pharmaceuticals (Basel). 2023 Oct 2;16(10):1398. doi: 10.3390/ph16101398 (PMC10609944; doi:10.3390/ph16101398)
Supplement: Supplementary file 1 [file pharmaceuticals-16-01398-s001.zip › pharmaceuticals-2601260-supplementary.pdf]

NMR and LCMS data

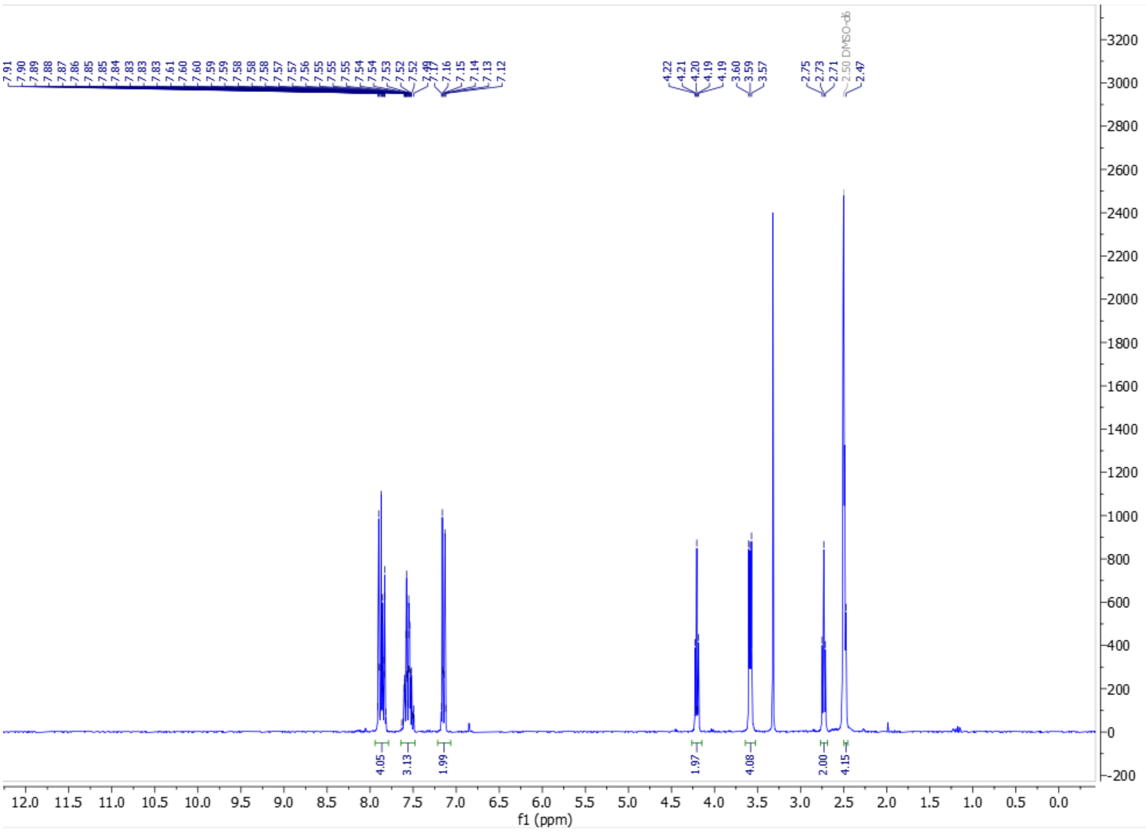

Figure S1. ^{1}H NMR (300 MHz, DMSO-d6) spectrum of compound **1** (ETH).

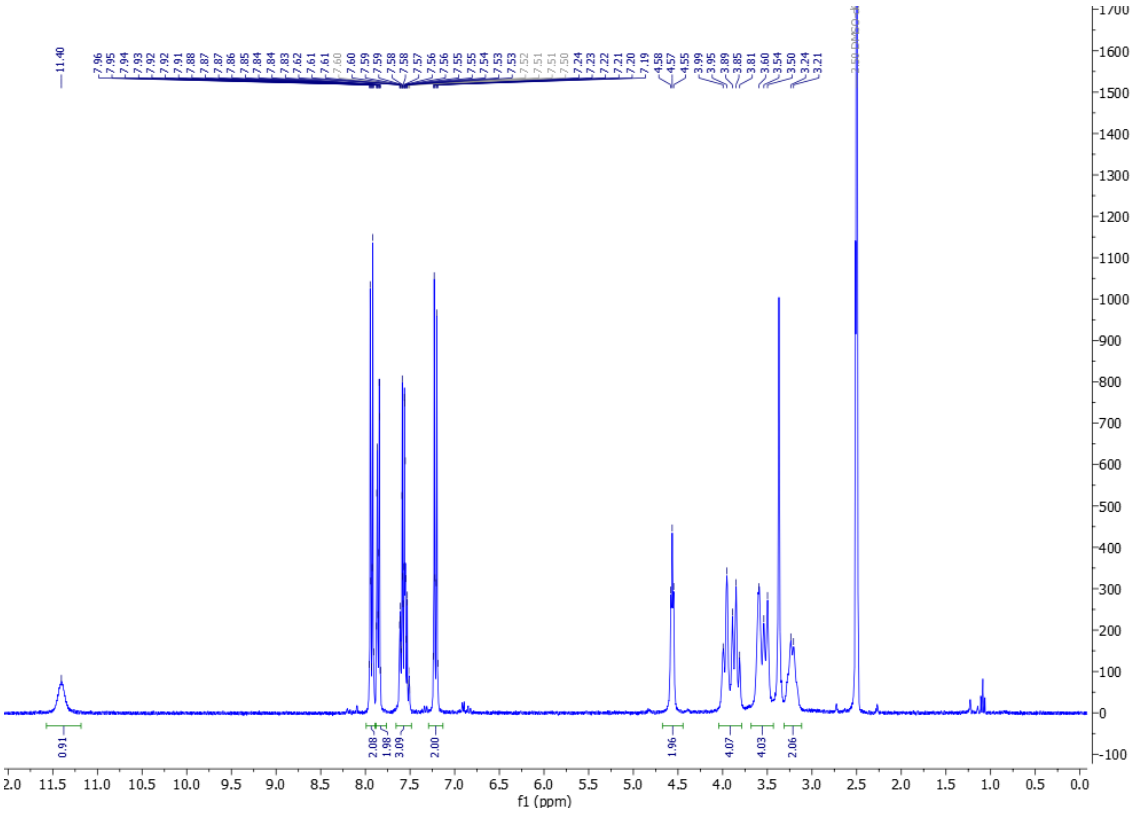

Figure S2. ^{1}H NMR (300 MHz, DMSO-d6) spectrum of compound **2** (ETH-HCl).

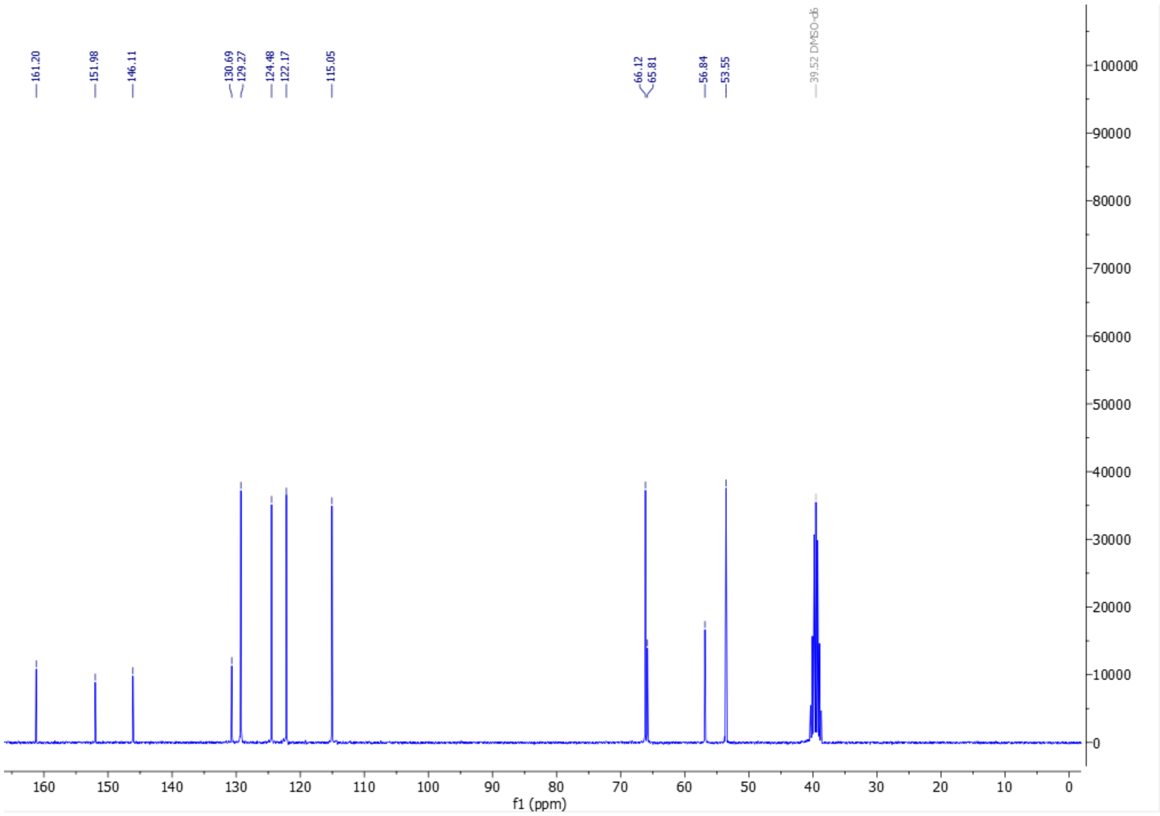

Figure S3. ^{13}C NMR (75 MHz, DMSO-d6) spectrum of compound **1** (ETH).

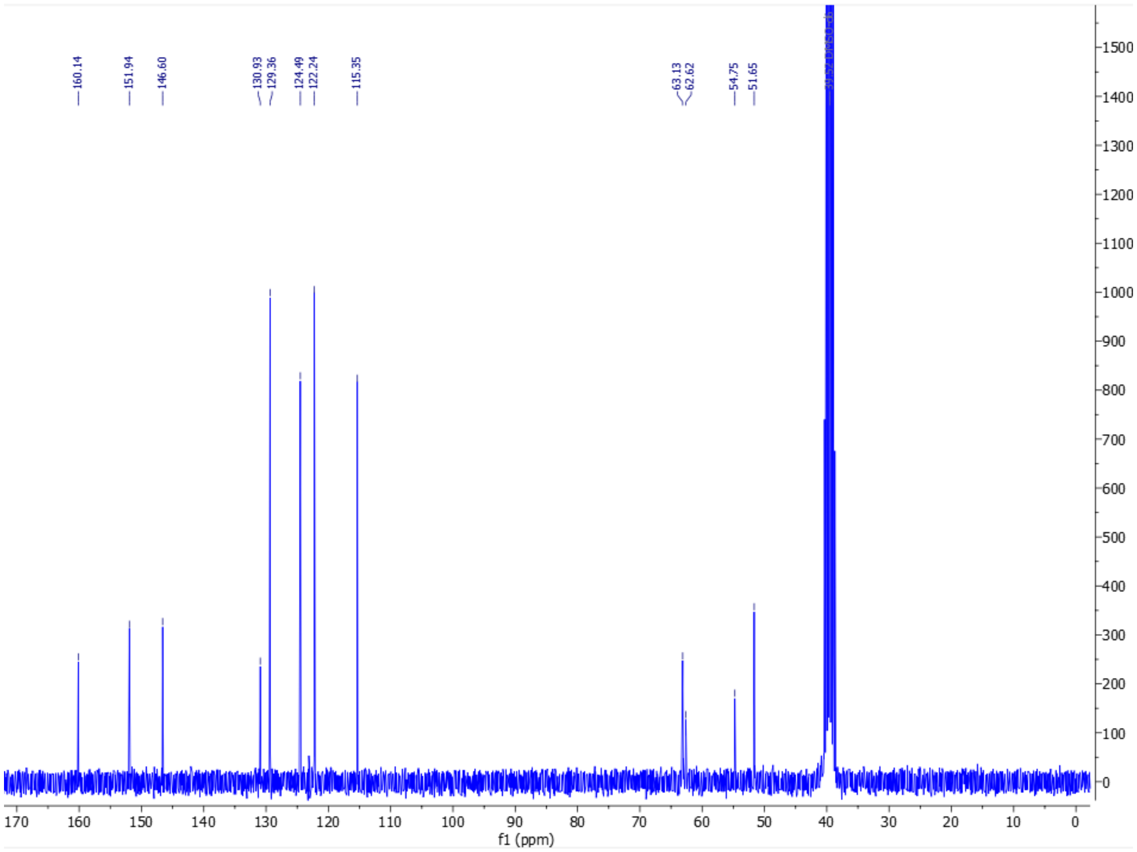

Figure S4. ^{13}C NMR (75 MHz, DMSO-d6) spectrum of compound **2** (ETH-HCl).

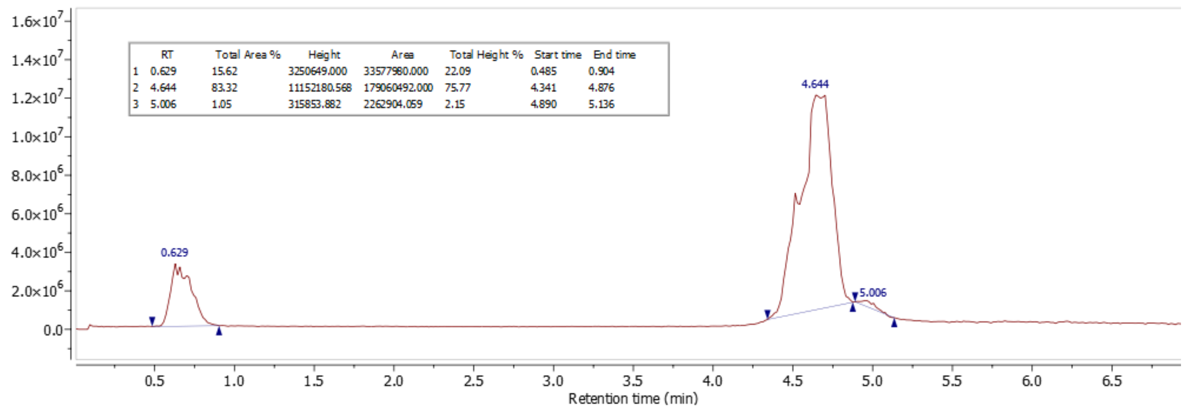

Figure S5. LC chromatogram for compound **2.** Two main peaks (0.63 min and 4.64 min corresponds to *Z* and *E* isomers respectively).

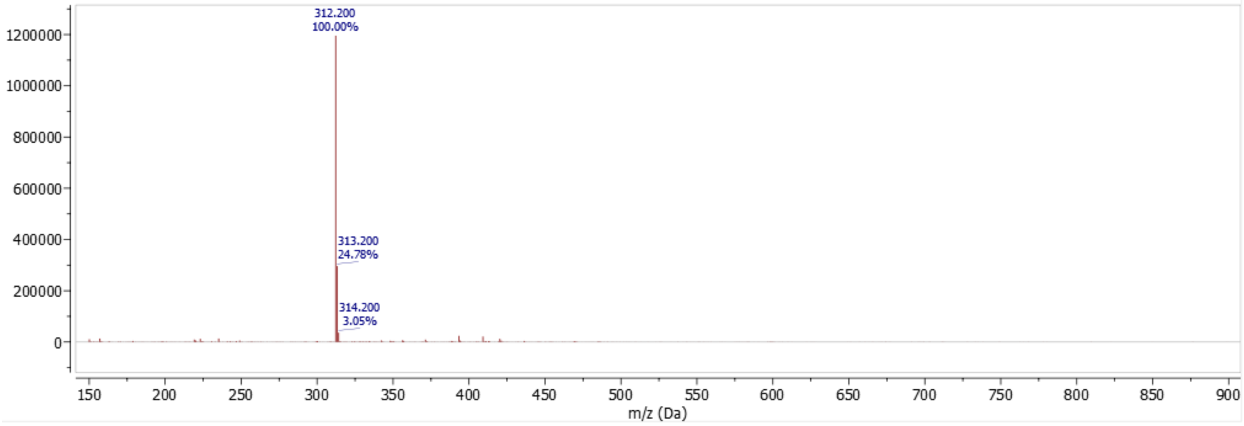

Figure S6. MS (ESI^+) spectrum of compound for peak 2 (RT=0.63 min, compound **2**, *Z*-isomer)

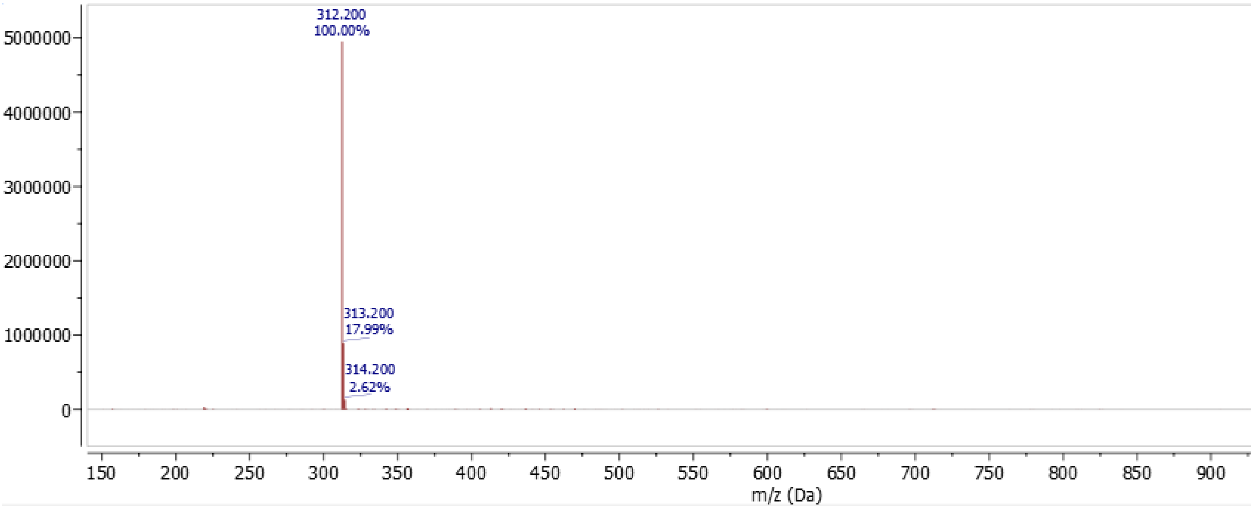

Figure S7. MS (ESI^+) spectrum of compound for peak 2 (RT=4.64 min, compound **2**, *E*-isomer)
